# Eclampsia with hypothyroidism complicated with posterior reversible encephalopathy syndrome–a case report

**DOI:** 10.1186/s12883-023-03068-y

**Published:** 2023-02-10

**Authors:** Xuejing Yin, Yu Duan, Lifang Zhang, Zhichao Feng, Caixia Yin, Sujie Zhu, Jinhua Chen, Xinsen Peng

**Affiliations:** 1 Department of Neurology, Changzhi Medical College, Changzhi, Shanxi Province 046000 China; 2 Department of Emergency Medicine, Changzhi People’s Hospital, The Affiliated Hospital of Shanxi Medical University, Changzhi, Shanxi Province 046000 China; 3 Department of Neurology, Changzhi People’s Hospital, The Affiliated Hospital of Shanxi Medical University, Changzhi, Shanxi Province 046000 China; 4 Department of Neurology, Shanxi Medical University, Taiyuan, Shanxi Province 030000 China; 5 Department of Emergency Medicine and Neurology, The Affiliated Hospital of Shanxi Medical University, No. 502 Changxing Middle Road, Changzhi, Shanxi Province 046000 China; 6 Department of Cardiovascular Medicine, Changzhi Medical College, Changzhi, Shanxi Province 046000 China

**Keywords:** Posterior reversible encephalopathy syndrome, Eclampsia, Hypothyroidism, Reversible cerebral vasoconstriction syndrome, Case report

## Abstract

**Background:**

Posterior reversible encephalopathy syndrome (PRES) is a rare neurological disorder with complex physiopathological mechanisms that have not been fully understood. Early identification is of great prognostic significance, of which the symptoms and radiological abnormalities can be completely reversed. If the diagnosis and treatment are delayed, ischemia and massive infarction may be developed in some patients. Posterior reversible encephalopathy syndrome (PRES) has been reported mainly in association with postpartum eclampsia, which have been rarely reported, while the association with hypothyroidism has not been reported at home or abroad.

**Case presentation:**

Here we report on a pregnant 29-year-old with multipara and a chief complication of hypothyroidism. She presented in the emergency department with frequent attacks of severe headache symptoms resulting from reversible cerebral vasoconstriction syndrome (RCVS), accompanied with prenatal eclampsia. PRES was determined by radiological examination.

**Conclusion:**

To the best of our knowledge, this is the first case of PRES complicated by hypothyroidism and prepartum eclampsia.Clinicians should be alert for the co-occurence of eclampsia, PRES, and RCVS when patients have convulsions after a typical throbbing headache. Moreover, regular monitoring of thyroid function during pregnancy should also occupy certain special attention.

## Background

Reversible cerebral vasoconstriction syndrome (RCVS) is characterized by severe thunderclap headache (TCH) and is most commonly observed in women aged 20–50 years [1]. The specific pathophysiological mechanism of RCVS remains to be elucidated, however it is proposed to manifest due to alterations in cerebrovascular tension induced by spontaneous trigger factors such as pregnancy, or exogenous trigger factors such as vasoactive drugs [2, 3].

Eclampsia, a rare and severe complication of the hypertensive disorder preeclampsia, is diagnosed in women who have reported one or more seizures before, during, or after childbirth [4]. Clinical features of eclampsia include systolic blood pressure ≥ 160 mmHg, proteinuria in the renal range (> 3.5 g/24 hours urine), renal impairment, thrombocytopenia and/or microangiopathic hemolytic anemia, hepatocyte injury, pulmonary edema, and neurological dysfunction [5]. Prior to an attack of eclampsia, one or more of the clinical manifestations are observed: headache, blurred vision, photophobia, abdominal pain and changes in mental state [e.g., decreased alertness] [6].

PRES was first reported by Hinchey et al. [7] in 1996. As it was initially regarded to only involve white matter, it was first termed reversible posterior leukoencephalopathy. However, when gray matter involvement was later confirmed, the term PRES was proposed [8]. PRES is a clinico-radiological syndrome primarily characterized by rapid onset of acute headache, vomiting, visual impairment, seizures, and sensory alterations [7, 8]. PRES is induced by multiple clinical conditions, such as cancer chemotherapy and autoimmune disease, but is commonly associated with acute hypertension and, in particular, preeclampsia and eclampsia [9, 10]. The main pathophysiological mechanism of PRES is indicated by hypertensive encephalopathy and vascular endothelial injury resulting from dysfunctional cerebrovascular autoregulation, and the emergence of cerebral vasospasm, tissue ischemia, and cerebral edema due to increased cerebrovascular permeability [11].

Hypothyroidism is associated with endothelial dysfunction secondary to decreased production of vasoactive substances, which could elicit impaired vasodilation, increased sympathetic tension and vascular resistance, eventually promoting pregnancy-induced hypertension and eclampsia [12].

All four of the aforementioned diseases are frequently identified during pregnancy, however RCVS and PRES tend to occur in the postpartum period. Interestingly, in the present case, all four diseases occurred simultaneously in the perinatal period, which, to the best of our knowledge, has not been previously reported.

## Case presentation

A 29-year-old female with pregnancy of 27 weeks presented at hospital with the chief complaint of “systemic edema for three days,headache for two days and vomiting for seven hours” on February 22,2022.She was transferred to the emergency department of our hospital. At 22:30, she developed limb spasms, trismus, foaming at the mouth, and loss of consciousness. Her blood pressure was 200/150 mmHg. An emergency intramuscular injection of magnesium sulfate 5 g was given and the consciousness was restored in 2 min and the other symptoms also resolve.

It was determined that she had PRES due to eclampsia, and she was admitted to the obstetrics department.Our diagnosis was based on the following presentation: Temperature, 36.1℃, Pulse Rate 120 bpm; Respiratory Rate 22 bpm, Blood Pressure 140/95 mmHg. She was consciousness, had normal visual field in both eyes, had negative meningeal stimulation sign, moderate edema in both lower limbs, abdominal distension, a uterine height of 23 cm, and an abdominal circumference of 108 cm. No obvious abnormality was found in the fetal ultrasonography. The fetal position was head first, and the fetal heart rate was 140 bpm. The patient’s clinical background showed no history of epilepsy or neurological disease and normal blood pressure during pregnancy. Hypothyroidism for the past four years was treated with oral Euthyrox (62.5 ug/day).

**Fig. 1:**
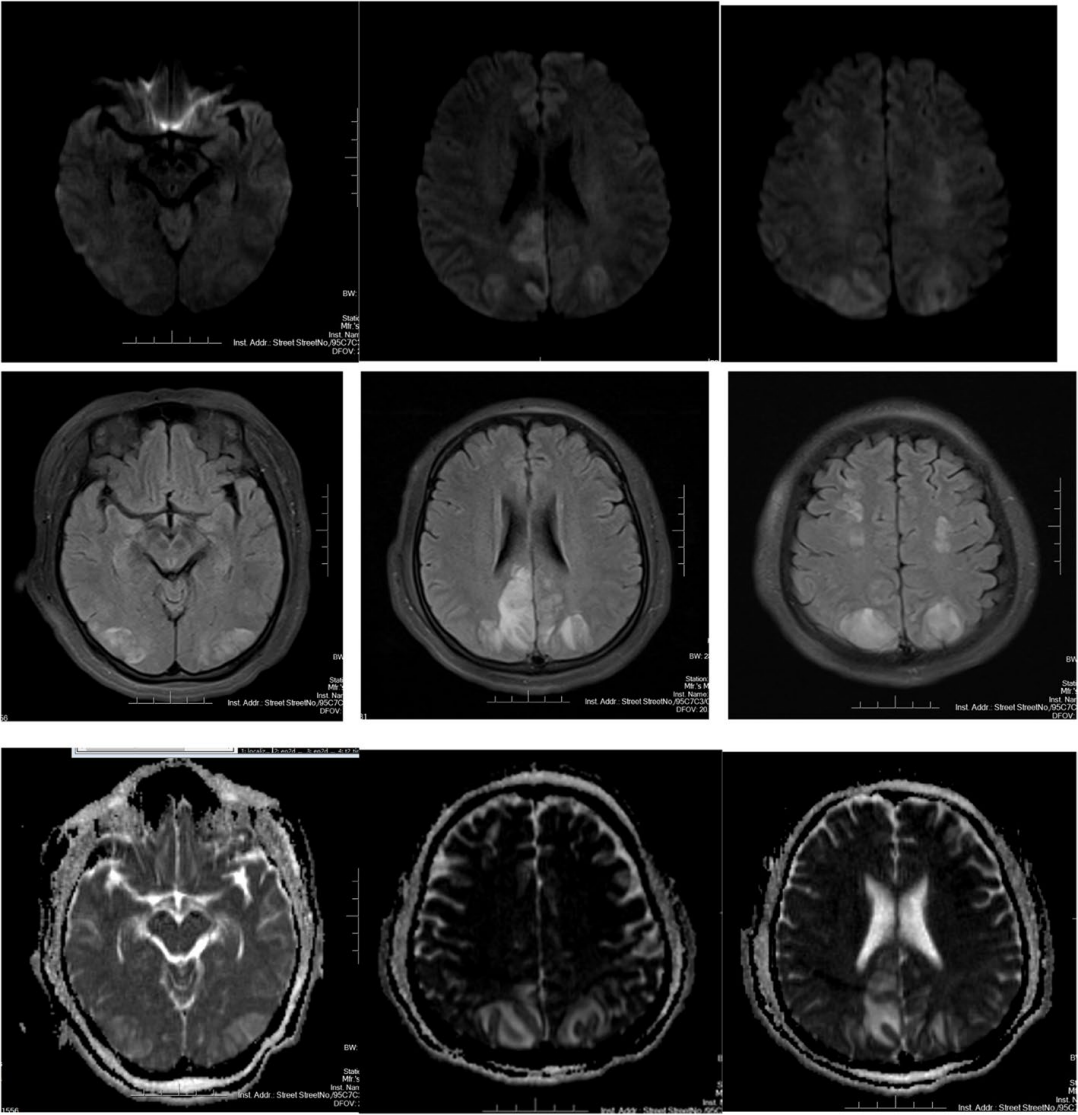
Brain MRI in a patient with Eclampsia with Hypothyroidism Complicated with Posterior Reversible Encephalopathy Syndrome (PRES). Legend: diffusion restriction on the diffusion-weighted sequences and apparent dispersion coefficient (Both sides)

Auxiliary examination using magnetic resonance imaging (MRI) fluid attenuated inversion recovery (FLAIR) showed multiple areas of flaky edema with high signal intensity in the bilateral frontoparietal cortex and subcortex. Hyperintensity was also observed in diffusion -weighted imaging (DWI) and apparent diffusion coefficient (ADC), and local gyri swelling (Fig. 1). Additional, multiple areas of flaky edema were observed with low-density foci in the bilateral parietal lobe computerised tomography (CT) scan.The patient was prescribed magnesium sulfate for prevention of eclamptic seizures, furosemide for diuresis, and dexamethasone and cefuroxime for anti-infection. Thyroid function examination indicated high thyroid stimulating hormone (TSH)、high C-reactive protein (CPR). No abnormalities were identified according to the dilated fundus examination. An emergency caesarean section was carried out after the assessment. Negative amniotic fluid (AF) culture was observed. However, the creatinine and brain natriuretic peptide(BNP) were seen to be increased on re-examination and impaired cardiac and renal functions was considered. In addition, mild hypertension (141/81 mmHg) was investegated after the caesarean. No neurological symptoms or convulsions were observed. Magnesium sulfate was continued to control the patient ‘s blood pressure and sedation, and furosemide was given for diuretic therapy. In order to prevent the patient’ s seizures again, sound and bright light stimulation were avoided as far as possible.

On the fifth day after the attack of eclampsia, the patient was discharged from the hospital. The typical PRES signs and symptomshad almost completely subsided according to the follow-up MRI, and blood pressure and thyroid function returned to within the normal range after delivery. The patient had no complaints of headache or further convulsions. (See Table 1 for laboratory tests during hospitalization).

**Table 1:** Laboratory data before and after delivery

	Day1	Day 2	Day 5	Day 18	Reference Range
WBC^1^, ×109/L	22.80	13.1	6.26	6.98	3.5–5.5
HGB, g/L	151	108	114	141	115–150
USG	1.030	1.044	1.012	1.016	1.003–1.03
PRO, g/L	> 3.0	10	1.0	0.15	0
KET, mmol/L	0.5	0	0	0	0
BLD, cells/Ul	200	200	25	0	(-)
WBC^2^, /HP	6–10	0–3	0	0	0–3
Ca, mmol/L	2.31	1.98	-	2.49	2.11–2.52
ALB, g/L	32.8	-	30.6	43.9	40–55
D-D, U/L	4.31	7.87	-	1.32	0-0.55
Fg, g/L	4.35	3.46	-	3.20	2–4
CK, U/L	168	356	-	160	40–200
CK-MB, U/L	34	238	-	85	0–25
LDH, U/L	388	299	-	235	120–250
BNP, pg/ml	1600	4534	1866	221	0-450
cTnT, pg/ml	116.2	-	-	54.3	0–14
Mb, ng/ml	172.8	-	-	82.1	28–72
UREA, mmol/L	6.0	-	3.00	5.00	2.6–7.5
UA, umol/L	622	641	280	343	155–357
CRE, umol/L	79.7	85.6	54.0	64.0	41–73

## Discussion

The clinical symptoms of PRES can be reversed by rapid treatment within a few days of onset, however a small number of patients may continue to show neurological deficits after treatment [13]. Neurologic symptoms occurring in pregnant women often indicates an increased risk for maternal and infant mortality [14]. As such, the risk of PRES induced by eclampsia is increasingly recognised.

Interestingly, owing to the COVID-19 pandemic, the number of reported PRES casessignificantly increased [15, 16]. It is known that COVID-19 adheres to ACE 2 receptors on endothelial cells and brain microglia could induce transformations in the renin-angiotensin-aldosterone system (RAAS) in favor of the classical pathway, thus leading to changes in vasoconstriction and in blood pressure, indirectly impacting on the cerebrovascular system leading to PRES. Due to the current high incidence of COVID-19, different types of neurological manifestations of COVID-19 have been increasingly and rapidly reported, covering those involving COVID-19, PRES and acute disseminated encephalomyelitis (ADEM) [15–17]. Therefore, the routine monitoring of COVID-19 in patients should be performed at admission to prevent misdiagnosis and missed diagnosis. In the present case, the patient was admitted to hospital unvaccinated with negative new coronavirus nucleic acid,therefore PRES induced by coronavirus was not considered for the time being.

An interesting observation in this case was that RCVS, which usually occurs during the delivery/postpartum period [18], occurred before eclampsia, which to the best of our knowledge has not previously been reported as a pre-eclampsia symptom until now. This suggests that a RCVS imaging examinations in pregnant women with severe headache before the attack of eclampsia may be conductive to early diagnosis [19]. Some studies have demonstrated that the early use of calcium channel blockers (CCBs, such as nimodipine) could contribute to stabilizing vasoconstriction and distal arteriole vasodilation in 64–83% of patients [13], however, this still remains a controversial treatment for prenatal eclampsia. Some suggest it is not recommended for pregnant women under 20 weeks of gestation on the grounds that it can reduce placental blood flow in maternal hypotension, eliciting changes in fetal heart rate [18, 20, 21]. Others suggest that it results in no significant changes in uterine or fetal arterial blood flow patterns [22], and the specific mechanism of action remains to be elucidated. It is widely acknowledged that magnesium sulfate can effectively prevent seizures in patients with eclampsia [23].Here, consciousness was rapidly restored within 2 min and blood pressure dropped to normal without visual field damage. Importantly, the prognosis was satisfactory following the emergency cesarean section.

In terms of the relationship between PRES and RCVS, we found that there are similarities between PRES and RCVS in terms of triggers, pathophysiological mechanisms, clinical presentation, and imaging features [24, 25]. Both PRES and RCVS are associated with ischemic and hemorrhagic manifestations and vasogenic edema can be seen on brain imaging. Furthermore, segmental vasoconstriction manifestations similar to RCVS can be seen in some PRES patients on angiography [24, 26].Although there are overlapping features in many aspects, there are still some cases of RCVS without vasogenic edema and PRES without vasoconstriction that cannot be explained according to our current understanding of the pathophysiological mechanisms, so it remains controversial as to PRES and RCVS are two independent overlapping individuals or different stages in the continuous process [27].

Studies have demonstrated the involvement of thyroid hormone in the regulation of placental development, endothelial function, and blood pressure. Furthermore, inflammatory mediators and thyroid hormone hormones significantly influence key processes of placental formation (decidua cell migration and angiogenesis) [28, 29]. Both hyperthyroidism and hypothyroidism are associated with adverse pregnancy outcomes, such as miscarriage, intrauterine growth retardation, preterm delivery, and pre-eclampsia [30, 31]. According to literature review, Hashimoto encephalopathy and [32] hyperthyroidism have been reported to be related to [33] PRES, while the specific mechanism remains unclear. Whether eclampsia lies the link between hypothyroidism and PRES requires to be further studied.

This case report has limitations. A CCB was not prescribed in the patient pre-cesarean, so it cannot be determined whether CCB can prevent eclampsia. In addition, RCVS may act as a prodromal symptom of eclampsia, and cranial vascular examination should be performed before and after the attack of eclampsia to determine whether the cerebrovascular changes were reversed. Importantly, magnetic resonance venography (MRV) should be actively examined to validate whether headache is induced by venous sinus thrombosis considering that thrombus may form in the perinatal state. Because the patient was very sick and had radiation concerns, we did not use CT venography. Clinically, more attention should be paid to patients with severe pulsing headache and pre-eclampsia or eclampsia, with simultaneous assessment of PRES and RCVS by brain imaging. Clinicians should be alert for the co-occurrence of eclampsia, PRES and RCVS when patients have convulsions after a typical throbbing headache. Moreover, regular monitoring of thyroid function during pregnancy should also be considered. Quick diagnosis and treatment can completely reverse patients’ symptoms and radiological abnormalities. If diagnosis and treatment are delayed, some patients will progress to ischemia, largescale infarction, or death. Therefore, further studies are required to elucidate the pathophysiological mechanisms underpinning co-occurrence of thyroid dysfunction, PRES, RCVS and eclampsia.

## Data Availability

The datasets and images used and analyzed during the current study are provided in the manuscript and available from the corresponding author on reasonable request.

## Abbreviations

PRES: Posterior reversible encephalopathy syndrome
RCVS: Reversible cerebral vasoconstriction syndrome
TCH: thunderclap headache
CCB: calcium channel blockers
RAAS: renin-angiotensin-aldosterone system
ADEM: Acute disseminated encephalomyelitis
MRI: Magnetic resonance imaging
DWI: Diffusion -weighted imaging
ADC: Apparent diffusion coefficient
CT: computerised tomography
MRV: Magnetic resonance venography
MRA: Magnetic resonance angiography
TSH: Thyroid stimulating hormone
CRP: C-reactive protein
BNP: Brain natriuretic peptide
WBC1: White blood cell
Hb: Hemoglobin
USG: Urine specific gravity
PRO: Urine protein
KET: Urine ketone
BLD: Urine blood
WBC2: Urine White blood cell
ALB: albumin
D-D: D-Dimer
Fg: Fibrinogen
CK: Creatine kinase
CK-MB: Creatine kinase isoenzymes
LDH: Lactic dehydrogenase
BNP: B-type natriuretic peptide
TnT: Troponin T
Mb: Myoglobin
UREA: Urea
UA: Uric acid
CRE: Creatinine

## References

[1] Burton TM, Bushnell CD (2019). Reversible cerebral vasoconstriction syndrome. Stroke.

[2] Miller TR, Shivashankar R, Mossa-Basha M, Gandhi D (2015). Reversible cerebral vasoconstriction syndrome, part 1: Epidemiology, Pathogenesis, and clinical course. AJNR Am J Neuroradiol.

[3] Miller TR, Shivashankar R, Mossa-Basha M, Gandhi D (2015). Reversible cerebral vasoconstriction syndrome, part 2: Diagnostic Work-Up, Imaging evaluation, and Differential diagnosis. AJNR Am J Neuroradiol.

[4] Which anticonvulsant for women with eclampsia? Evidence from the Collaborative Eclampsia Trial. Lancet. 1995;345(8963):1455–63. 7769899

[5] Lindheimer MD, Taler SJ, Cunningham FG, American Society of Hypertension (2009). ASH position paper: hypertension in pregnancy. J Clin Hypertens (Greenwich).

[6] Hypertension in pregnancy (2013). Report of the American College of Obstetricians and Gynecologists’ Task Force on hypertension in pregnancy. Obstet Gynecol.

[7] Hinchey J, Chaves C, Appignani B (1996). A reversible posterior leukoencephalopathy syndrome. N Engl J Med.

[8] Dillon WP, Rowley H (1998). The reversible posterior cerebral edema syndrome. AJNR Am J Neuroradiol.

[9] Fugate JE, Rabinstein AA (2015). Posterior reversible encephalopathy syndrome: clinical and radiological manifestations, pathophysiology, and outstanding questions [published correction appears in Lancet Neurol. 2015 Sep;14(9):874]. Lancet Neurol.

[10] Gao B, Lyu C, Lerner A, McKinney AM (2018). Controversy of posterior reversible encephalopathy syndrome: what have we learnt in the last 20 years?. J Neurol Neurosurg Psychiatry.

[11] Shankar J, Banfield J (2017). Posterior reversible Encephalopathy Syndrome: a review. Can Assoc Radiol J.

[12] Toloza FJK, Derakhshan A, Männistö T (2022). Association between maternal thyroid function and risk of gestational hypertension and pre-eclampsia: a systematic review and individual-participant data meta-analysis. Lancet Diabetes Endocrinol.

[13] Pirker A, Kramer L, Voller B, Loader B, Auff E, Prayer D (2011). Type of edema in posterior reversible encephalopathy syndrome depends on serum albumin levels: an MR imaging study in 28 patients. AJNR Am J Neuroradiol.

[14] Fischer M, Schmutzhard E (2017). Posterior reversible encephalopathy syndrome. J Neurol.

[15] Koralnik IJ, Tyler KL (2020). COVID-19: A global threat to the nervous system. Ann Neurol.

[16] Pons S, Fodil S, Azoulay E, Zafrani L (2020). The vascular endothelium: the cornerstone of organ dysfunction in severe SARS-CoV-2 infection. Crit Care.

[17] Cheng H, Wang Y, Wang GQ (2020). Organ-protective effect of angiotensin-converting enzyme 2 and its effect on the prognosis of COVID-19. J Med Virol.

[18] Tanaka K, Matsushima M, Matsuzawa Y (2015). Antepartum reversible cerebral vasoconstriction syndrome with pre-eclampsia and reversible posterior leukoencephalopathy. J Obstet Gynaecol Res.

[19] Ueno S, Takeda J, Maruyama Y, Makino S, Miyamoto N, Itakura A (2020). Antepartum eclampsia with reversible cerebral vasoconstriction and posterior reversible encephalopathy syndromes. J Obstet Gynaecol Res.

[20] Chen SP, Fuh JL, Wang SJ (2011). Reversible cerebral vasoconstriction syndrome: current and future perspectives. Expert Rev Neurother.

[21] Cho S, Lee MJ, Chung CS (2019). Effect of Nimodipine Treatment on the clinical course of reversible cerebral vasoconstriction syndrome. Front Neurol.

[22] Grzesiak M, Ahmed RB, Wilczynski J (2013). 48-hours administration of nifedipine in spontaneous preterm labor - doppler blood flow assessment of placental and fetal circulation. Neuro Endocrinol Lett.

[23] Burton GJ, Redman CW, Roberts JM, Moffett A (2019). Pre-eclampsia: pathophysiology and clinical implications. BMJ.

[24] Pilato F, Distefano M, Calandrelli R (2020). Posterior reversible encephalopathy syndrome and reversible cerebral vasoconstriction syndrome: clinical and radiological considerations. Front Neurol.

[25] Fugate JE, Rabinstein AA (2015). Posterior reversible encephalopathy syndrome: clinical and radiological manifestations, pathophysiology, and outstanding questions. Lancet Neurol.

[26] Lee MJ, Cha J, Choi HA (2017). Blood-brain barrier breakdown in reversible cerebral vasoconstriction syndrome:implications for pathophysiology and diagnosis. Ann Neurol.

[27] Jeanneret V, Jillella DV, Rangaraju S (2022). PRES and RCVS: two distinct entities or a spectrum of the same disease?. J Stroke Cerebrovasc Dis.

[28] Adu-Gyamfi EA, Wang YX, Ding YB (2020). The interplay between thyroid hormones and the placenta: a comprehensive review†. Biol Reprod.

[29] Silva JF, Ocarino NM, Serakides R (2014). Maternal thyroid dysfunction affects placental profile of inflammatory mediators and the intrauterine trophoblast migration kinetics. Reproduction.

[30] Taylor PN, Lazarus JH (2019). Hypothyroidism in pregnancy. Endocrinol Metab Clin North Am.

[31] Korevaar TIM, Medici M, Visser TJ, Peeters RP (2017). Thyroid disease in pregnancy: new insights in diagnosis and clinical management. Nat Rev Endocrinol.

[32] Kato Y, Nakazato Y, Ito Y, Tomioka R, Tamura N, Shimazu K. Rinsho Shinkeigaku. 2006;46(8):550–4. 17154034

[33] Cagney D, Razzaq Z, Majeed M, O’Leary DP, Redmond HP (2021). Primary hyperparathyroidism causing posterior reversible encephalopathy syndrome: a case report. Ann R Coll Surg Engl.

